# Complete Genome Sequence of *Limosilactobacillus reuteri* Strain VHProbi E18, Isolated from Feces of an Old Man

**DOI:** 10.1128/mra.01211-21

**Published:** 2022-05-18

**Authors:** Hongchang Cui, Zhi Duan

**Affiliations:** a Qingdao Vland Biotech Group Co., Ltd., Qingdao, China; Universidad Nacional Autónoma de México

## Abstract

*Limosilactobacillus reuteri* strain VHProbi E18 is a Chinese commercial lactic acid bacterium with several probiotic functions. Here, we report the whole-genome sequence of this bacterium. The whole-genome sequencing yielded a 2,040,678-bp genome and 2,015 protein-coding genes.

## ANNOUNCEMENT

Fecal samples collected from a healthy old man were inoculated in de Man-Rogosa-Sharpe (MRS) agar medium and incubated for 48 h in an anoxic chamber at 37°C. A bacterial strain was designated E18. Characterization was performed by 16S rRNA Sanger sequencing, mass spectrometry, and microbiology methods. The results showed that the E18 strain belongs to the species Limosilactobacillus reuteri. The culture was deposited in the China Center for Type Culture Collection (CCTCC) (CCTCC number M2021153). A combined strategy with Illumina HiSeq 2500 and Pacific Biosciences (PacBio) RS II sequencing technology was used to sequence the whole genome of L. reuteri strain VHProbi E18. Total DNA was extracted using a genomic DNA purification kit (Promega, USA) following the manufacturer's instructions. Illumina template DNA was sheared into 400-bp fragments for library creation using a TruSeq library preparation kit from Illumina. Illumina sequencing libraries were prepared from the sheared fragments using the NEXTflex rapid DNA sequencing (DNA-Seq) kit (Bioo Scientific, USA). For PacBio sequencing, an aliquot of 15 μg DNA was centrifuged in a g-TUBE (Covaris, MA) at 6,000 rpm for 60 s using an Eppendorf 5424 centrifuge. DNA fragments were then purified, end repaired, and ligated with SMRTbell sequencing adapters (PacBio, CA) following the manufacturer’s recommendations. The resulting sequencing library was purified three times using a 0.45 volume of Agencourt AMPure XP beads (Beckman Coulter Genomics, MA) following the manufacturer’s recommendations. Next, an ~10-kb insert library was prepared and sequenced on one single-molecule real-time (SMRT) Cell using standard methods. Finally, 7,448,802 raw reads and 7,289,670 clean reads were generated to reach a depth of 1,157.08-fold coverage using the Illumina sequencing platform; 283,864 raw reads were generated using the PacBio RS II sequencing platform. The Illumina-generated sequence reads were quality filtered by Sickle v1.33 (https://github.com/najoshi/sickle), and low-quality reads were removed before assembly. The PacBio read *N*_50_ value was 12,608 bp. Genome assembly was performed using SOAPdenovo v2.04 (1). The PacBio raw reads were then assembled into a contig using Unicycler v0.4.8 (2). Pilon v1.22 was used for the performance of error correction of the PacBio assembly results (3). The last circular step was checked manually. If there was an overlap at both ends of the final assembly sequence with a certain length, then the sequence was circularized and the overlap sequence was cut off one end to obtain a complete genome sequence and plasmid sequence. Glimmer v3.02 (4), tRNA-scan-SE v2.0 (5), and barrnap v0.9 (https://github.com/tseemann/barrnap) were used to predict protein-coding genes, tRNAs, and rRNAs, respectively. CheckM v1.1.3 was used to check the quality of the assembled genome sequence, with completeness of 99.45%, contamination of 0%, and strain heterogeneity of 0% (6). Genome annotation was conducted using the NCBI Prokaryotic Genome Annotation Pipeline (PGAP) v5.3 (7). Default parameters were used for all software unless otherwise specified.

The final assembly yielded a single contig, and the complete genome sequence of L. reuteri VHProbi E18 contains a circular 2,040,678-bp chromosome with a GC content of 38.88%. There were 1,938 coding genes, 18 rRNA genes (5S rRNA, 16S rRNA, and 23S rRNA genes), 69 tRNA genes, 3 noncoding RNA genes, and 48 pseudogenes in the genome.

### Data availability.

The complete genome sequence of L. reuteri VHProbi E18 has been deposited in the GenBank database with accession number CP088931, SRA accession numbers SRR17617303 and SRR18531191, BioProject accession number PRJNA785080, and BioSample accession number SAMN23526821.

## References

[1] Luo R, Liu B, Xie Y, Li Z, Huang W, Yuan J, He G, Chen Y, Pan Q, Liu Y, Tang J, Wu G, Zhang H, Shi Y, Liu Y, Yu C, Wang B, Lu Y, Han C, Cheung DW, Yiu S-M, Peng S, Xiaoqian Z, Liu G, Liao X, Li Y, Yang H, Wang J, Lam T-W, Wang J. 2012. SOAPdenovo2: an empirically improved memory-efficient short-read de novo assembler. Gigascience 1:18. doi:10.1186/2047-217X-1-18. 23587118 PMC3626529

[2] Wick RR, Judd LM, Gorrie CL, Holt KE. 2017. Unicycler: resolving bacterial genome assemblies from short and long sequencing reads. PLoS Comput Biol 13:e1005595. doi:10.1371/journal.pcbi.1005595. 28594827 PMC5481147

[3] Walker BJ, Abeel T, Shea T, Priest M, Abouelliel A, Sakthikumar S, Cuomo CA, Zeng Q, Wortman J, Young SK, Earl AM. 2014. Pilon: an integrated tool for comprehensive microbial variant detection and genome assembly improvement. PLoS One 9:e112963. doi:10.1371/journal.pone.0112963. 25409509 PMC4237348

[4] Ingram S, Munzner T, Olano M. 2009. Glimmer: multilevel MDS on the GPU. IEEE Trans Vis Comput Graph 15:249–261. doi:10.1109/TVCG.2008.85. 19147889

[5] Chan PP, Lowe TM. 2019. tRNAscan-SE: searching for tRNA genes in genomic sequences. Methods Mol Biol 1962:1–14. doi:10.1007/978-1-4939-9173-0_1. 31020551 PMC6768409

[6] Parks DH, Imelfort M, Skennerton CT, Hugenholtz P, Tyson GW. 2015. CheckM: assessing the quality of microbial genomes recovered from isolates, single cells, and metagenomes. Genome Res 25:1043–1055. doi:10.1101/gr.186072.114. 25977477 PMC4484387

[7] Tatusova T, DiCuccio M, Badretdin A, Chetvernin V, Nawrocki EP, Zaslavsky L, Lomsadze A, Pruitt KD, Borodovsky M, Ostell J. 2016. NCBI Prokaryotic Genome Annotation Pipeline. Nucleic Acids Res 44:6614–6624. doi:10.1093/nar/gkw569. 27342282 PMC5001611

